# Restoring indigenous names in taxonomy

**DOI:** 10.1038/s42003-020-01344-y

**Published:** 2020-10-23

**Authors:** Len Norman Gillman, Shane Donald Wright

**Affiliations:** 1 Faculty of Design and Creative Technology, Auckland University of Technology, St Paul Street, Auckland, 1010 New Zealand; 2 School of Biological Sciences, University of Auckland, 20 Symonds Street, Auckland, 1010 New Zealand

**Keywords:** Plant sciences, Zoology, Scientific community

## Abstract

Some pillars of scientific practice appear immutable. We propose that one of these needs more thorough consideration and modification: this being the long-standing emphasis in nomenclature for first published names over pre-existing indigenous names, in accepting species epithets. We suggest that biologists re-evaluate this practice, in the context of a current more general re-evaluation of indigenous knowledge. We propose that it is now time to critically examine taxonomic protocols in favour of both assigning and reinstating indigenous names whenever possible.

## Abstract (web summary)

Gillman and Wright propose a re-evaluation of taxonomical nomenclature to reinstate indigenous species names. These authors discuss the consideration of indigenous names for new and existing species in order to reflect the importance and precedence of indigenous knowledge in biology.

The species name is a fundamental unit. However, for Indigenous Peoples, the name in its vernacular form may also embody history, a sense of place and a right to belong. Like the Latin binomial, indigenous names for plants and animals can also be knowledge conduits. When Europeans colonised ‘new’ lands, they often claimed possession merely by a proclamation of discovery and deposed local geographic place names with new ones. In a similar way, biologists have introduced new species names with nomenclature publications that often set aside long-standing indigenous names. There are, however, exceptions, and examples of indigenous names having been used in the binomial include among trees; *Beilschmiedia tawa*, (A.Cunn.) Benth. et Hook.f. ex Kirk in New Zealand, *Couratari tauari* Berg. in Amazonia and *Colophospermum mopane* (Kirk ex Benth.) Kirk ex J. Leonard in southern Africa. More generally, authorities in many countries are reinstating indigenous names for localities and geographic features and the scientific community is now recognising indigenous knowledge particularly for ecosystem management^1,2^. Taxonomists are also increasingly engaging in consultation with Indigenous Peoples over names and using indigenous language for newly described or assigned taxa^3,4^. Here, we advocate for a broader shift in practice involving changes to the rules of taxonomic codes.

## Discussion

The protocols and rules for nomenclature are established and maintained by scientific societies^5,6^: researchers, editors, land managers, together with authors of field guides, popular publications and websites, then accommodate these rules in the form of applied usage. Fundamental to this system is priority. However, the almost ubiquitous chronological precedence of indigenous names has no standing or priority under current taxonomic codes, despite these often conveying in-depth knowledge relating to form, uses, distribution and ecology.

One value of the scientific binominal is in reducing confusion between common names. Nonetheless, ongoing taxonomic revision often results in the accumulation of many synonyms. In such cases the system does little to reduce the confusion of common names. The application of the principle of conservation under current codes has reduced the number of name changes but they still occur. This flux in nomenclature has often prevailed in the face of constancy in an indigenous vernacular name. For example, the species name for the New Zealand forest tree *Prumnopitys taxifolia* (D.Don) de Laub. has had five epithets (one of which includes the indigenous name of *matai*), whereas the indigenous vernacular name of mataī has remained unchanged for centuries. In the case of *Colophospermum mopane*, the long-term use of the indigenous “mopane” for the species epithet has fortunately remained stable during generic shifts in nomenclature. Additionally, there is a plethora of disparate species with the same epithet. For example, *colensoi* applies to at least 19 plant species, two bird species and two fungi in New Zealand. This, despite many of these species having well recognised indigenous names that could be applied and thereby help to avoid the homogenisation rendered by such duplication.

An approach to nomenclature that might be described as ‘colonial’ was reflected in the earlier penchant for assigning surnames that were habitually erected to honour collectors, sponsors, colleagues or employers who were often distanced from the country in question. In total this previous effort currently defines a large proportion of the nomenclatural diaspora. Further, some epithets from the past are rather insensitive to place and historical circumstance such as, for example, *Melidectes whitemanensis* Gilliard as the binomial for a honeyeater endemic to New Britain in the heart of Melanesia. Another example derives from the colloquial and pejorative name of “kaffir” in southern Africa which has subsequently been applied to a number of edible plant species. We believe it is unfortunate that the word has also been incorporated into some binomials; for example, the edible fruit tree species, *Dovyalis caffra* Warb. and *Harpephyllum caffrum* Bernh. ex C. Krauss. Species epithets, such as the examples above, convey no morphological nor ecological information. Instead, they merely recall outdated thinking that seems rather odd in a more pluralistic contemporary setting. These are names that are now likely to have little resonance for biologists in the country of origin and that may be, at best, irrelevant, and at worst, offensive, to the resident Indigenous Peoples. By contrast, there is a latent opportunity for species epithets, and indeed for names at any taxonomic level, to reflect their importance for Indigenous People. For example, one of the most important fish genera for food among Amazonia’s inhabitants is *Cichla*^7^, with the widely recognised vernacular name ‘tucunare’. Either the genus or one of the eight recognised species could carry that identity and this would affirm its importance to the Indigenous People in the region.

We envisage taxonomic rule changes to promote retrospective name changes that establish, on the basis of precedence, pre-existing indigenous names for species where possible. The first step in this process should be a general debate on the merits or otherwise of a new approach. This debate must include Indigenous Peoples and indigenous scientists as prominent stakeholders in the discussion. We hope that this letter will act as a catalyst for such debate and that support will build around the idea.

The next phase would be to submit formal proposals to the governing bodies of nomenclature for rule changes. Nomenclature is governed, among others: by the International Code of Nomenclature for algae, fungi, and plants (ICN) and proposed rule changes can be submitted via publication in *Taxon*, or ‘from the floor’ by anyone attending the nomenclature sessions^3^; and by the International Commission on Zoological Nomenclature (ICZN), the latter of which considers submissions for amending the code under its constitution^4^.

Under the ICN, article 11.4 states that “for any taxon below the rank of genus, the correct name is the combination of the final epithet of the earliest legitimate name of the taxon at the same rank …”. This clause establishes priority for earliest legitimate names but it also includes exceptions to the rule such as for the conservation of long established names. Our proposal would include a further exception whereby indigenous names can replace previously legitimate names on the basis of their actual chronological precedence, both in discovery and usage, as opposed to the priority afforded under existing nomenclature rules. Article 12.1 requires “valid publication” for a name to have status. An exception to this rule would therefore need to be included to allow indigenous names to have status under the code following submission by Indigenous Peoples. Such submissions would need to present evidence that the taxon in question has a dominantly applied indigenous vernacular name across its distribution. Additional articles would be needed to establish the criteria by which this status could be achieved. These criteria would need to include: (1) how to evaluate the relationship between the Indigenous People making the submission and the species under consideration and whether or not there are competing nomenclatural interests; (2) evidence of a consensus across the indigenous community covering a geographic area that included the whole distribution of the species under consideration. Where different groups of Indigenous Peoples are unable to form a consensus over the preferred name for a particular taxon then no indigenous name would have status and the taxon name would remain unchanged.

Under the ICZN, Article 23 sets out the principle of priority whereby the “valid name of a taxon is the oldest available name applied to it”. In a similar manner proposed above for plants, Article 23 under the ICZN would need to be amended to provide an exception to allow the earlier indigenous names to indeed be deemed the oldest available name applied to a taxon (following submission by the relevant Indigenous People[s]). Again, additional articles would need to be added setting out criteria for this exception. Throughout both codes minor amendments would be required to provide alignment with the new provisions. It should be recognised that, in making these changes, any conceptual challenge to such alteration occurs in the context of a right deriving from the temporal precedence of indigenous nomenclature.

## Conclusion

The number of taxa across the globe that could be considered for such retrospective name changes will be limited, but the changes we propose would herald an important step in the affirmation of Indigenous People’s contribution to nomenclature and knowledge.

Thus, we envision a new order in which names such as the New Zealand forest tree species, *Prumnopitys taxifolia* would become *Prumnopitys matai*, the east African riparian tree species, *Breonadia salicina* (Vahl) Hepper & J. R. I. Wood would become *Breonadia matumi*, and the North American edible deciduous forest tree, *Diospyros virginiana* L. would become *Diospyros pessamin*. Such a legacy could become axiomatic under a new respect for indigenous precedence in nomenclature.

## References

[1] Ban NC (2018). Incorporate Indigenous perspectives for impactful research and effective management. Nat. Ecol. Evol..

[2] Lyver POB (2016). Key biocultural values to guide restoration action and planning in New Zealand. Restor. Ecol..

[3] Veale AJ (2019). Using te reo Māori and ta re Moriori in taxonomy. N.Z. J. Ecol..

[4] Nielsen SV, Bauer AM, Jackman TR, Hitchmough RA, Daugherty CH (2011). New Zealand geckos (Diplodactylidae): cryptic diversity in a post-Gondwanan lineage with trans-Tasman affinities. Mol. Phylogenet. Evol..

[5] Turland, N. et al. *International Code of Nomenclature for algae, fungi, and plants (Shenzhen Code) adopted by the Nineteenth International Botanical Congress Shenzhen, China, July 2017*. (Koeltz Botanical Books, 2018).

[6] Ride, W. D. et al. *International code of zoological nomenclature*. 4th edn, (International Trust for Zoological Nomenclature, The Natural History Museum, 1999).

[7] van der Sleen, P. & Albert, J. S. *Field Guide to the Fishes of the Amazon, Orinoco, and Guianas*. (Princeton University Press, 2018).

